# Abusive supervision and nursing students’ intention to leave the nursing profession: a moderated mediation model of emotional exhaustion and the nurse-patient relationship

**DOI:** 10.1186/s12912-024-02025-5

**Published:** 2024-05-30

**Authors:** Youjuan Hong, Meijing Chen, Caimei Chen, Meichai Qiu

**Affiliations:** 1 School of Nursing, Fujian Medical University, Fuzhou, China; 2 Critical Care Department, Longyan People’s Hospital, Longyan, Fujian China; 3 Center for information Management, Fujian Provincial Hospital, Fuzhou, Fujian China

**Keywords:** Abusive supervision, Emotional exhaustion, Nurse-patient relationship, Future work intention, Clinical learning environment

## Abstract

**Background:**

Exploration of the relationship between nursing students’ abusive supervision and their future intention to leave the nursing profession before completing the final clinical practicum is critical to the issue of nursing staff shortages and how to alleviate them. In order to further dissect the factors influencing turnover intention among student nurses in clinical practice, our study used the conservation of resources theory and job demands-resources model to explain the specific pathways that influence student nurses’ intention to leave the nursing profession, with particular focus on nursing students’ personality traits and certain organizational factors.

**Method:**

This study followed a cross-sectional design. Between March and May 2022, a convenience sampling method was used to select 531 nursing students from two medical universities in Fuzhou. The Abusive Supervision, Emotional Exhaustion, Nurse-Patient Relationship, and Turnover Intention Scales were employed to collect data. The PROCESS macro (Models 4 and 7) for SPSS 25.0 by Hayes and 5,000 bootstrap samples were used to examine the moderation and mediation impacts.

**Results:**

Abusive supervision was found to significantly positively predict nursing students’ intention to leave the nursing profession. Emotional exhaustion significantly mediated the relationship between abusive supervision and an intention to leave the nursing profession. The moderating effect of the nurse-patient relationship in the mediation model was also found to be significant.

**Conclusions:**

Abusive supervision by clinical teaching staff is a work-related stressor that leads to emotional exhaustion, consequently decreasing nursing students’ future intention to work as a nurse. A nurse-patient relationship based on trust could buffer the negative effect of abusive supervision on emotional exhaustion. Healthcare organizations and nurse educators should implement programs that educate and train individuals about abusive supervision, emotion regulation, and positive nurse-patient relationships; this would serve to decrease nursing students’ intention to leave the nursing profession. This study provides relevant implications for helping nursing instructors develop effective intervention strategies to retain talented nursing personnel.

## Introduction

Nursing shortages have become a pressing issue worldwide [1, 2]. There are only 2.7 nurses per 1,000 people nationwide in China, less than half the average worldwide and far below the ratio in other developed countries such as the United States (8.3) and Switzerland (17.5). Lin (2020) found that as many as 45.4% of nursing students in China intend to leave the nursing profession [3]. Nursing students abandoning the nursing profession upon graduation exacerbates nursing workforce shortfalls, increases the financial burden on healthcare organizations [4], reduces the quality of patient care [5], and decreases patient satisfaction [6]. Understanding the influential factors and underlying mechanisms are especially important for identifying ways to reduce turnover among student nurses.

The final clinical practicum (FCP), which occurs just before graduation, provides a real-world context through which nursing students can acquire clinical skills; it can also help graduating students develop a positive attitude regarding their future nursing careers [7]. Nonetheless, nursing students often report dissatisfaction with their clinical placements, and the result is an intention to leave [8]. Several past studies have found that newly graduated nurses’ turnover intention was influenced by factors such as authentic leadership, organizational identification, occupational coping self-efficacy [9], empowerment [10], work-life fit, and work-life interference [11]. Explorations of abusive supervision have indicated that it is a prevalent problem in healthcare organizations [12, 13]. However, little research has focused on the effects of abusive supervision among nursing students during their FCP. Reducing stress induced by abusive supervision will improve the retention of nursing students, a change that is vital for medical organizations. In addition, very few research studies have sought to investigate the moderated mediating functions operating between abusive supervision and turnover intention, particularly those moderators that could buffer the harmful effects of abusive supervision on student nurses’ turnover intention. Therefore, the potential relationship between abusive supervision and student nurses’ turnover intention during the FCP has yet to be analyzed.

The present research builds upon the literature and expands our understanding of the negative consequences of abusive supervision in relation to nursing students’ intention to work as a nurse after graduation. Abusive supervision is a negative leadership construct that has numerous adverse effects [14, 15]. We drew upon the conservation of resources theory (COR) to explore emotional exhaustion as a potential mechanism for understanding why abusive supervision might lead to nursing students’ intention to leave the nursing profession [16]. We also examined the perceived nurse-patient relationship’s ability to act as a moderator, buffering the negative effects of abusive supervision on turnover intention due to its ability to supply the resources needed by nursing students.

## Background

### Abusive supervision and nursing students’ intention to leave the nursing profession

Abusive supervision is defined as subordinates’ perceptions of their supervisor’s display of sustained hostile verbal or nonverbal behavior, including public criticism, rudeness, breaking promises, humiliation, and the “silent treatment” [17]. Previous studies have found abusive supervision to correlate with job satisfaction, psychological strain [18], psychological health [19], and an increased intent to quit [20]. Before graduating, nursing students must spend one year in a hospital internship to turn theoretical knowledge into skills, thus qualifying them to become nurses after graduation. Supervisors are nursing professionals who teach and monitor students; they play an important role in the nursing students’ onboarding process and influence their adjustment progression [21, 22]. Negative experiences with supervisors have been associated with an intention to leave the nursing profession [23]. Abusive supervision may occur when a supervisor intends to show power over or control a subordinate. Favaro, Wong, and Oudshoorn (2021) found that younger and less experienced nurses of both genders experienced greater bullying from supervisors [10].

As new employees in a health organization, the quality of nursing students’ relationship with their supervisors is an important heuristic for making inferences about their connection with the health organization [22]. The more supervisors adopt abusive behavior, the more perceived injustice emerges. Nursing students may come to feel that their work is not recognized by their supervisors or they are not worthy of the organization [24]. In addition, student nurses experiencing this form of abuse tend to have bad relationships with their supervisors and feel that they have no concern for their wellbeing. Thus, abusive supervision is a work-related stressor that can lead to the voluntary or involuntary termination of employees [25]. One study found that abusive supervision increased nurses’ intention to leave their organization [18]. If they were not in contact with an abusive supervisor, their job satisfaction was enhanced and intention to leave reduced.

### Emotional exhaustion as a mediator

Wright and Cropanzano (1998) defined emotional exhaustion as “a chronic state of physical and emotional depletion caused by excessive job demands and ongoing hassles” [26]. Abusive behavior by superiors is a persistent source of work stress that can result in emotional exhaustion and burnout [27]. Employees’ anxiety, despondency, and emotional exhaustion increase in parallel with their access to an aggressive supervisor. This is because when employees face abusive behavior from their superiors, they become agitated, perceive a threat to their identity and social position, and in certain situations, experience moral outrage [15].

Emotional exhaustion occurs when emotional demands exceed an individual’s coping potential, in this case due to interpersonal workplace interactions [28]. The COR can be used to explain the mediating mechanism through which abusive supervision affects turnover intention [16]. The theory posits that individuals have an intrinsic tendency to gain, preserve, and protect their resources. Therefore, nursing students experiencing emotional stress due to abusive supervision will fear losing resources, run the risk of doing so, and likely have trouble replacing them. As a result, they must exert additional psychological effort when faced with abusive supervision [29]. As a consequence of exerting this extra mental effort to deal with abusive superiors, nursing students’ emotional reserves are depleted, exacerbating their emotional fatigue. Without these resources, nursing students become dissatisfied and may decide to leave the profession [30]. According to previous research, emotional exhaustion significantly predicts both the intention to leave the nursing profession and actual turnover [19, 31].

### Nurse-patient relationship as a moderator

The nurse-patient relationship is a work-oriented, interpersonal, caregiving relationship that nurses establish with patients and their families through nursing activities [32]. Previous research has found that a bad nurse-patient relationship is positively correlated with low job satisfaction [33], high turnover intention [34], perceived stress, and reduced job involvement [35]. In contrast, a positive nurse-patient relationship aids nurses in terms of their socioemotional needs. Employees in a positive nurse-patient relationship are likely to conserve the emotional resources needed to survive and function in the workplace. Thus, according to the job demands-resources model (JDR), a positive nurse-patient relationship is a job resource that contributes to the alleviation of job burnout and role stress. Employees’ emotional exhaustion can vary in relation to differences in their perceived nurse-patient relationship. A better relationship positively affects nurses’ work status; the better the relationship, the more willing nurses are to engage fully in clinical work, thus improving patient health outcomes. Therefore, a good nurse-patient relationship is an important resource, helping employees handle the detrimental impacts of stress and stay focused on their roles [32]. For instance, Jiang (2022) found that the impact of stressors on emotional exhaustion tends to be strong for nurses who have a tense nurse-patient relationship [36]. Therefore, a positive nurse-patient relationship can directly provide resources effective for reducing the emotional exhaustion caused by abusive supervision. For example, one previous study found that patients’ lack of reciprocity moderated the positive relationship between abusive supervision and mental health risks among nurses [37]. Therefore, a positive nurse-patient relationship is likely to buffer the connection between abusive supervision and emotional exhaustion. Specifically, the influence of abusive supervision on emotional exhaustion is likely to be weaker for those with positive nurse-patient relationships. Thus, we propose the following:

### The present study: goals and hypotheses

**Fig. 1:**
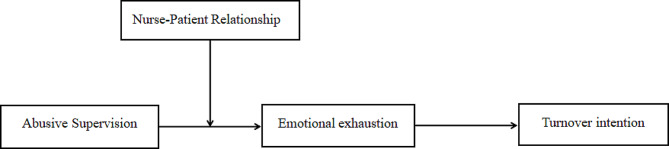
Proposed moderated mediation model

Drawing upon the COR and JDR, the present study explores why and when abusive supervision are associated with turnover intention in student nurses. Based on a literature review, we propose a moderated mediation model where the indirect effect of abusive supervision on turnover intention via emotional exhaustion depends on the level of positivity in the perceived nurse-patient relationship. The hypothesized moderated mediation model is presented in Fig. 1 and comprised of the following three hypotheses:

#### Hypothesis 1

Abusive supervision is positively correlated with nursing students’ intention to leave the nursing profession.

#### Hypothesis 2

Emotional exhaustion mediates the relationship between abusive supervision and an intention to leave the nursing profession.

#### Hypothesis 3

The perceived nurse-patient relationship moderates the relationship between abusive supervision and emotional exhaustion.

## Method

### Participants

This research utilized a quantitative cross-sectional methodology. Data collection occurred between March and May 2022. It was mandatory that participants be senior nursing students participating in their last clinical practicum. All had or were in the process of completing a clinical practicum lasting more than six months and had been paired with one registered nurse instructor. A total of 590 participants responded to a cross-sectional research questionnaire. There were 531 valid questionnaires obtained after excluding those that were invalid (59 had missing values for abusive supervision, emotional exhaustion, and/or turnover intention). The participants included 410 females (77.21%), and the average age was 21.67 years (SD = 0.68). According to Bentler and Chou [38], the sample size should be more than 10 times the observed variables. Thus, a sample size of 531 met the requirement for further analysis.

### Procedure

Surveys were distributed to participants from two medical universities in southern China. After obtaining informed consent and emphasizing the anonymity of responses, participants completed the questionnaires on the Wenjuanxing website platform, which is a professional online questionnaire survey platform in China. Participants answered all the measurements in the Chinese language.

### Ethical considerations

#### Ethical approval

was granted by the Ethics Committee of Fujian Medical University. Informed consent was obtained during the initial stage of the study, prior to survey completion. All participants were informed of the study’s goal, assured of confidentiality and anonymity, and informed that they could withdraw at any moment and for any reason.

#### Measurements

##### The abusive supervision scale

Abusive supervision was assessed using the Abusive Supervision Scale [39]. This scale has five items rated on a five-point Likert scale ranging from 1 (strongly disagree) to 5 (strongly agree). An example item is as follows: “My supervisor put me down in front of others during my internship.” Higher scores on the scale indicate higher levels of abusive supervision. The Cronbach’s alpha for this scale in the present study was 0.91, showing good internal consistency.

### The emotional exhaustion scale

Emotional exhaustion was assessed using the Emotional Exhaustion Scale [40]. This scale has six items rated on a 5-point Likert scale ranging from 1 (strongly disagree) to 5 (strongly agree). An example item is as follows: “I feel very tired every morning when I think about facing a whole day’s work.” Higher scores on the scale indicate higher levels of emotional exhaustion. In our study this scale showed good internal consistency, with a Cronbach’s alpha of 0.88.

### The nurse-patient relationship scale

The nurse-patient relationship was measured using the Chinese version of the Nurse-Patient Relationship Scale [41]. This scale has four items rated on a 5-point Likert scale ranging from 1 (strongly disagree) to 5 (strongly agree). Higher scores indicate a better nurse-patient relationship. An example item is as follows: “I feel like patients treated me with respect during my internship.” In the present study, the Cronbach’s alpha for this scale was 0.76.

### The turnover intention scale

The intention to leave the nursing profession was measured using the Chinese version of the Turnover Intention Scale [42]. This scale has four items rated on a 5-point Likert scale ranging from 1 (strongly disagree) to 5 (strongly agree). An example item is as follows: “I often think about leaving my current career.” Higher scores indicate a greater willingness to leave. In the present study, the Cronbach’s alpha for this scale was 0.74.

### Statistical analysis

SPSS 26.0 and SPSS PROCESS v4.1 were used for the statistical analysis. First, descriptive analysis was used to describe the general demographic characteristics. Second, a Pearson’s correlation was applied to determine the correlations among abusive supervision, emotional exhaustion, nurse-patient relationship, and turnover intention. The issue of common method variance (CMV) was also verified using a Harman’s one-factor model [43]. Finally, the mediating role of emotional exhaustion operating between abusive supervision and turnover intention was first evaluated through the PROCESS macro (Model 4) of SPSS. In addition, the PROCESS macro (Model 7) was used to test the moderation of the nurse-patient relationship. The total, direct, and indirect effects of the model were evaluated and found to be statistically significant. If the 95% bootstrap confidence interval did not contain zero, a 95% bias-corrected CI based on 5,000 bootstrap samples was used.

## Results

### Common method variance test

The data collected were self-reported, and thus could have caused a common method bias problem. In order to reduce the risk of common method bias, a Harman one-way factor analysis was conducted to determine the presence of common method bias. The findings indicated that only 31.67% of the variation was explained by a single component, which is less than the critical value of 40%. Thus, there was no serious common method bias problem in this study.

### Sample characteristics

A total of 590 student nurses were initially included in this survey. Of these, 59 were then excluded due to missing data. The sex ratio of males to females was 1:29.51 and average age 21.67 ± 0.68 years. The average level of turnover intention was 12.28 ± 2.66. The details are shown in Table 1.

**Table 1:** Demographics Variables of the Student Nurses (*N* = 531)

	Frequency(%)
**Age**	
20	53(9.98%)
21	139(26.78%)
22	275(51.80%)
23	64(12.05%)
**Gender**	
Male	121(22.79%)
Female	410(77.21%)
**Place**	
City	145(27.31%)
Countryside	386(72.69%)

### Descriptive statistics

Means, standard deviations, and correlations among the variables are shown in Table 2. The results show that abusive supervision was positively correlated with emotional exhaustion (*r* = 0.56, *p* < 0.01) and an intention to leave the nursing profession (*r* = 0.48, *p* < 0.01) and negatively related to the nurse-patient relationship (*r* = -0.48, *p* < 0.01), supporting Hypothesis 1. The results also show that emotional exhaustion was positively correlated with an intention to leave the nursing profession (*r* = 0.58, *p* < 0.01). Moreover, the nurse-patient relationship was negatively related to emotional exhaustion (*r* = -0.53, *p* < 0.01) and an intention to leave the nursing profession (*r = -*0.47, *p* < 0.01).

**Table 2:** Descriptive Statistics

Variable	M	SD	1	2	3	4
1. Abusive supervision	1.65	0.69	-			
2. Emotional exhaustion	3.05	0.86	0.56^***^	1		
3. Turnover Intention	2.75	0.79	0.48^***^	0.58^***^	1	
4.Nurse-patient relationship	3.30	0.63	-0.48^***^	-0.53^***^	-0.47^***^	1

Note: M = mean; SD = standard deviation. ^*^p < 0.05, ^**^p#x2009;< 0.01, ^***^p < 0.001

### Mediation of emotional exhaustion

The SPSS PROCESS macro Model 4 was used to examine the mediating role of emotional exhaustion. After controlling for gender and age, abusive supervision was found to significantly predict an intention to leave the nursing profession (Model 1: *β* = 0.42, *p* < 0.001) and emotional exhaustion (Model 2: *β* = 0.57, *p* < 0.001). Moreover, emotional exhaustion was significantly correlated with an intention to leave the nursing profession (Model 3: *β* = 0.54, *p* < 0.001), and the direct association between abusive supervision and an intention to leave the nursing profession remained significant (Model 3: *β* = 0.15, *p* < 0.05). The bias-corrected percentile bootstrap analyses showed that emotional exhaustion partially mediated the relationship between abusive supervision and an intention to leave the nursing profession (indirect effect = 0.31, Boot SE = 0.04, 95% CI = [0.23, 0.39]). The contribution rate of the mediating effect to the total effect was 67.84%. Therefore, Hypothesis 2 was supported (see Table 3).

**Table 3:** Testing for a Mediation Effect

Predictor	Model 1 (Turnover Intention)		Model 2 (Emotional Exhaustion)		Model 3 (Turnover Intention)	
	*β*	*t*	*β*	*t*	*β*	*t*
Gender	0.03	0.02	0.06	0.58	0.02	0.19
Age	0.52	0.02	-0.01	-0.10	0.03	0.31
Abusive Supervision	0.42^***^	8.57	0.57^***^	15.60	0.15^**^	3.59
Emotional Exhaustion					0.54^***^	13.52
*R* ^*2*^	0.18		0.31		0.40	
*F*	24.64^***^		49.73^***^		54.71^***^	

^*^p < 0.05, ^**^p < 0.01, ^***^p < 0.001

### Moderation of the nurse-patient relationship

In order to examine Hypothesis 3, interaction effects were analyzed with the PROCESS macro Model 7. The results show that abusive supervision had a positive predictive effect on an intention to leave the nursing profession (*β* = 0.40, *p* < 0.001); the interaction effect of abusive supervision and nurse-patient relationship on emotional exhaustion was significant (*ß* = 0.13, *p* < 0.001), and emotional exhaustion had a positive predictive effect on an intention to leave the nursing profession (*ß* = 0.54, *p* < 0.001). These findings indicate that the nurse-patient relationship moderated the association between abusive supervision and emotional exhaustion. Hypotheses 3 was supported (see Table 4).

**Table 4:** Coefficients for the Tested Moderated Mediation Model

Predictor	Model 1 (Emotional Exhaustion)			Model 2 (Turnover Intention)		
	*β*	*SE*	*t*	*β*	*SE*	*t*
Gender	0.01	0.09	0.05	0.01	0.09	0.01
Age	-0.05	0.08	-0.73	0.01	0.07	0.19
Abusive Supervision	0.40***	0.04	10.22	0.15**	0.05	3.59
Nurse-Patient Relationship	-0.37***	0.04	-9.41			
Abusive Supervision× Nurse-Patient Relationship	0.13***	0.04	3.12			
Emotional Exhaustion				0.54***	0.04	13.51
*R* ^*2*^	0.41			0.40		
*F*	44.22***			54.81***		

Note: × represents the interaction item of Abusive Supervision and Nurse-Patient Relationship

^*^p < 0.05, ^**^p < 0.01, ^***^p < 0.001

**Fig. 2:**
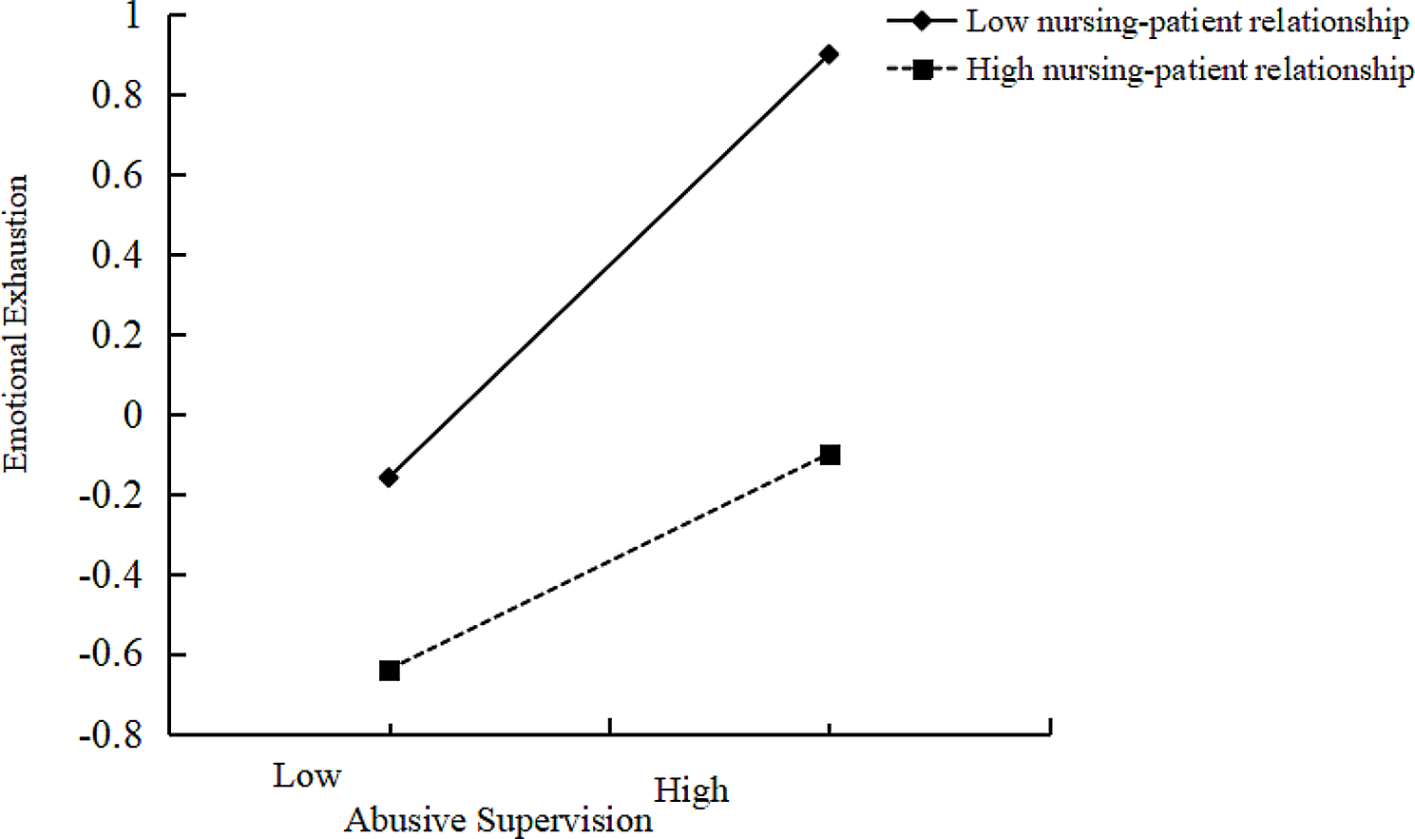
Interaction between abusive supervision and nurse-patient relationship

Additionally, a simple slope analysis was conducted to analyze the moderating effect of the nurse-patient relationship. Figure 2 illustrates this effect at two moderator levels: low (M – SD) and high (M + SD). For participants in the high and low nurse-patient relationship groups, abusive supervision had a positive effect on emotional exhaustion. In addition, for participants with a high nurse-patient relationship, the effect of abusive supervision on turnover intention (*β* = 0.27, *p* < 0.001) was weaker than for those in the low group (*β* = 0.51, *p* < 0.001), confirming our hypothesis that a high nurse-patient relationship weakens the positive relationship between abusive supervision and turnover intention. Specifically, as abusive supervision increased, emotional exhaustion increased more markedly in the low nurse-patient relationship group than in the high.

## Discussion

The goal of this study was to examine the effect of abusive supervision on an intention to leave the nursing profession among nursing students engaged in their FCP, as well as explore the mediating role of emotional exhaustion and moderating effect of the nurse-patient relationship. The results support our belief that abusive supervision increases students’ intention to leave the nursing profession, and that the relationship is mediated by emotional exhaustion. In this process, the nurse-patient relationship was found to play a moderating role. Specifically, the effect of abusive supervision on turnover intention in the high nurse-patient relationship group was found to be weaker than what was exhibited by the low group.

The results show that abusive supervision was positively correlated with an intention to leave the nursing profession, as well as that the former directly influencing the latter. Previous studies have confirmed the effect of abusive supervision on an intention to leave the nursing profession [44–46]. However, to the best of our knowledge, this is the first study to explore the effect of abusive supervision on an intention to leave the nursing profession among nursing students engaged in their FCP; this is essential in order to highlight the critical role of the supervisor (i.e., clinical teaching staff) in enhancing student nurses’ retention. Abusive supervision may pose a threat to student nurses and make them insecure; they may perceive themselves as being treated as outgroup members [47]. Thus, consistent pressure from supervisors is likely to push student nurses towards negative reactions such as an intention to leave the nursing profession [48]. The results indicate that student nurse supervisors play an important role in building a positive or negative workplace climate, which can in turn either strengthen or harm students’ workplace sense of belonging. Therefore, abusive supervision is costly, as it results in an increase in an intention to leave the nursing profession.

Our results also show that abusive supervision positively but indirectly influenced student nurses’ intention to leave the nursing profession, and emotional exhaustion played a mediating role [11]. In other words, abusive supervision can augment emotional exhaustion in student nurses and thus increase their likelihood of forming an intention to leave the profession, a result that can be explained by the COR [49]. Abusive supervision is a form of workplace stress that drains student nurses’ mental resources. When such students work in a stressful environment caused by their supervisor’s behavior, they may experience a threat to their resources and feeling of tiredness, resulting in high emotional exhaustion [50]. According to the COR theory, individuals seek to obtain, retain, and protect their resources and minimize the threat of resource loss [51]. Thus, emotionally exhausted student nurses may feel helpless, decreasing their desire to remain in the nursing profession. Consequently, emotional exhaustion was shown to be a strong predictor of an intention to leave among the student nurses studied. These results support the notion that abusive supervision has a broader impact on newcomers (in this context, nursing students engaged in clinical practice), not only within companies but also in medical organizations.

Moreover, the results also show that the nurse-patient relationship moderated the linkage between abusive supervision and emotional exhaustion. Though the influence of abusive supervision on turnover intention has been confirmed in previous studies [47, 52], there has been little research exploring the factors that might buffer its effects. To our knowledge, this is the first to provide empirical evidence of the effect of the nurse-patient relationship on the correlation between abusive supervision and an intention to leave the nursing profession among nursing students engaged in clinical practice. Therefore, it is a novel addition to the extant literature. The results are consistent with the JDR model [53]. Positive nursing-patient relationships are a job resource that contributes to the alleviation of role stress. An increase in the quality of the nurse-patient relationship was found to weaken the predictive effect of abusive supervision on emotional exhaustion. Student nurses with negative nurse-patient relationships rated stronger negative reactions to abusive supervision than did individuals with more positive relationships. Patient respect and trust is an important resource to help student nurses handle the detrimental impacts of stress and stay focused on their role [31]. Previous studies have focused more on the impact of poor nurse-patient relationships on an intention to leave the nursing profession, while this study confirmed the positive impact of positive nurse-patient relationships on positive psychological resources for nursing students engaged in clinical practice.

### Implications

Our results will improve the greater understanding of the organizational and individual factors affecting student nurses’ intention to leave the nursing profession, thus assisting instructors with developing effective intervention strategies to retain talented nursing personnel. First, the results highlight key practical implications for healthcare organizations seeking to avoid the negative consequences of abusive supervision by clinical practice supervisors. Nurturing positive relationships among supervisors and nursing students, as well as mutual respect and support, and creating an ideal clinical learning environment are central to decreasing nursing students’ voluntary turnover. Healthcare organizations should adopt these findings and implement programs designed to educate, train, and support clinical teaching staff to avoid abusive supervision. Second, nurse educators should incorporate emotion regulation into their specific intervention strategies for handling emotional exhaustion resulting from abusive supervision. Third, a patient-nurse relationship based on trust has critical importance for the intention to remain. Such relationships allow student nurses to feel respected and trusted by their patients. Nurse educators should develop training to improve nurse-patient communication and trust skills for nursing students.

### Limitations

There are several limitations of the current study that need to be considered. First, a cross-sectional design was adopted, and this restricted the analysis to the causal relationship between abusive supervision and an intention to leave the nursing profession. Longitudinal research should be conducted in the future, particularly as longitudinal studies are rarely adopted in the area of abusive supervision in clinical practice. Second, the data were collected through the self-reporting of nursing students via an online survey, which may have led to response bias based in social desirability. Other measurements should be adopted to draw stronger conclusions about the relationship between abusive supervision and its consequences. Third, this study did not explore the effects of different types of abusive supervision on the intention to leave the nursing profession. Different types of abusive supervision should be distinguished to confirm what particular types of abusive supervision can be appraised as most stressful.

## Conclusion

This study represents a first look at abusive supervision in healthcare organizations, using a sample of student nurses in China. We proposed and tested a novel model for abusive supervision’s direct, indirect, and relaxation effects on the intention to leave the nursing profession among nursing students engaged in clinical practice. The present study’s findings emphasize the important role of abusive supervision, emotional exhaustion, and nurse-patient relationships in nursing students’ intention to leave the profession. The results indicate that abusive supervision significantly affects nursing students’ intention to leave. Emotional exhaustion is also significantly related to nursing students’ intention to leave. Furthermore, emotional exhaustion indirectly affects the relationship between abusive supervision and nursing students’ intention to leave the nursing profession. This research adds to this research domain by examining the moderating role of nurse-patient relationships based on trust and confirms that such nurse-patient relationships could moderate the association between abusive supervision and an intention to leave the nursing profession. Consequently, patient support may enable student nurses to feel cared about, significant, and capable, and thus help eliminate the harmful effects of abusive supervisors for those engaged in the FCP.

## Data Availability

The data supporting the findings of this study can be obtained from the corresponding author upon reasonable request.
